# Automated 3D Quantitative Analysis of Intrapulmonary Vessel Volume on Non-contrast CT in Healthy Individuals

**DOI:** 10.2174/0115734056354924241115102310

**Published:** 2025-01-24

**Authors:** Ying Ming, Yu Zhang, Ran Xiao, Ruijie Zhao, Jiaru Wang, Sirong Piao, Lan Song, Yinghao Xu, Xin Sui, Wei Song

**Affiliations:** 1 Department of Radiology, Peking Union Medical College Hospital, Chinese Academy of Medical Sciences and Peking Union Medical College, Beijing 100730, China; 2 Research and Development Center, Canon Medical Systems (China), No.3, Xinyuan South Road, Chaoyang District, Beijing 100027, China; 3 CT Business Unit, Canon Medical Systems (China), No.3, Xinyuan South Road, Chaoyang District, Beijing 100027, China

**Keywords:** Lung, Computed tomography, Blood vessels, Window technique, Three-dimensional, Quantitative analysis

## Abstract

**Objective:**

This study aimed to compare automated three-dimensional Intrapulmonary Vessel Volume (IPVV) differences between lung and mediastinal windows in healthy individuals using quantitative measurements obtained from chest Computed Tomography (CT) plain scans.

**Methods:**

A total of 258 participants (aged 21–83 years) with negative chest CT scans from routine physical examinations conducted between January to November 2023 were retrospectively enrolled. For each healthy participant, an algorithm was used to automatically extract total lung IPVVs as well as IPVVs for vessels of specific diameter. Differences in IPVVs were then compared between those extracted using the lung window and those extracted using the mediastinal window.

**Results:**

The IPVVs for the entire lung, intrapulmonary arteries, intrapulmonary veins, and small pulmonary vessels (categorized by different diameters) extracted from the lung window were significantly higher than those extracted from the mediastinal window (*p*<0.01). No significant sex-based differences in IPVV were observed for pulmonary arteries and veins with diameters between 0.8 and 1.6 mm, as well as pulmonary veins with diameters between 2.4 and 3.2 mm. However, in pulmonary arteries and veins with diameters between 1.6 and 2.4 mm, females had significantly higher IPVVs than males. In all other cases, IPVVs were larger in males than in females.

**Conclusion:**

This method of automatic IPVV extraction and quantitative assessment has been proven to be feasible. Automated IPVV expression effectively identified morphological characteristics of intrapulmonary vessels. The study has concluded IPVVs extracted from the lung window to be generally larger than those extracted from the mediastinal window.

## 1 INTRODUCTION

Studying pulmonary vasculature morphology in healthy individuals is essential for understanding the complex network underpinning pulmonary functions [1]. Diseases that cause abnormal vascularization in the lungs impact both cardiac and pulmonary function. Pulmonary vasculature injury and reductions in pulmonary vessel volume can impair cardiac filling [2]. Existing studies on normal values for pulmonary vessel size and number have largely relied on autopsy findings, which have inherent limitations [3-5]. Consequently, there is a pressing need for more objective, non-invasive, and accurate methods to assess Intrapulmonary Vascular Volume (IPVV).

Computed Tomography (CT) is the most commonly used imaging modality for diagnosing and evaluating pulmonary diseases [6, 7]. CT scans can visualize small pulmonary vessels, assessing features, such as dilatation, tortuosity, and tapering in vessels that are only 1 mm in diameter [8, 9]. Sato *et al*. [10] and Frangi *et al*. [11] showed that pulmonary vessels have tube-like structures, and substantial research has focused on extracting and analyzing pulmonary vascular morphology. Numerous studies have proposed methods for segmenting pulmonary vessels in chest CT images [12]. However, arteries and veins are rarely analyzed separately because of technical limitations [13]. Identifying pulmonary arteries and veins remains challenging due to the high number of vessels, making manual labeling time-intensive and difficult to standardize. Computational algorithms, however, can independently identify intrapulmonary vessels from chest CT images and interpret related morphological information [7, 14], facilitating accurate identification and measurement of individual vascular segments.

Extracting and analyzing pulmonary vessels could aid in the non-invasive diagnosis of lung diseases and may be valuable for accessing, diagnosing, and predicting outcomes in patients with a history of smoking [15], pulmonary hypertension [13, 16, 17], Chronic Obstructive Pulmonary Disease (COPD) [18, 19], and/or Coronavirus Disease 2019 (COVID-19) [20, 21]. To date, however, no studies have examined potential discrepancies in quantitatively extracted IPVVs derived from either lung or mediastinal CT windows in healthy participants. Most previous studies have used the mediastinal window to measure vessel diameters [22].

In this study, we aimed to automatically extract IPVVs using a new post-processing method to separately analyze pulmonary arteries and veins and investigate differences in IPVV derived from lung and mediastinal windows *in vivo*. This non-invasive and quantitative evaluation tool may facilitate further research.

## 2 MATERIALS AND METHODS

### 2.1 Participants

This retrospective study, approved by the ethics committee and the institutional review board (no. I-23PJ2181), was granted an exemption from informed consent. A total of 317 individuals who underwent non-contrast chest Computed Tomography (CT) at our hospital between January and November 2023 were retrospectively enrolled.

The inclusion criteria were as follows: (1) clear images suitable for accurate segmentation, (2) negative CT chest plain scans, (3) no history of pulmonary disease, and (4) no history of dust exposure. Exclusion criteria included (1) a history of thoracic surgery, (2) inability to identify the algorithm due to poor-quality images, (3) pulmonary space-occupying lesions and severe pulmonary disease, including consolidations, pulmonary nodules, severe emphysema, and severe interstitial pneumonia, which could impact results, and (4) thoracic deformity.

Clinical metrics, including the height (cm) and weight (kg) of the participants, were collected from electronic medical records. All images were saved in Digital Imaging and Communications in Medicine (*DICOM*) format. Fig. (**1**) shows the flow diagram of the included individuals.

## 3 EXPERIMENTAL

### 3.1 CT Image Acquisition

All individuals underwent whole-lung volume CT scans. Non-contrast chest CT scans were performed using the IQon CT (Philips Medical Systems, Best, Netherlands) and Aquilion ONE GENESIS (Toshiba Medical Systems, Tokyo, Japan). Participants were instructed to hold their breath before the CT scan and during the examination and take on a supine position, supporting their heads with both arms. Breath-holding was performed at the end of deep inspiration, while whole-lung scans were performed from the pulmonary apex to the lung base. CT scan parameters were as follows: tube voltage, 120 kVp; tube current, automatic tube current modulation technology; rotation time, 0.5 s; image matrix, 512×512; collimation, 64×0.625 mm (IQon CT), 80×0.5 mm (Aquilion ONE GENESIS); field of view, < 410 mm. Instead of using complex iterative algorithms for noise reduction, the images were reconstructed using deep learning reconstruction (Advanced Intelligent Clear-IQ Engine, AiCE).

### 3.2 Quantitative Evaluation of Pulmonary Vessels

All image files in DICOM format were transferred to a separate post-processing workstation. An algorithm (Canon Medical Systems, Tokyo, Japan) automatically extracted and segmented pulmonary vessels with high repeatability. This process involved utilizing a pulmonary artery/vein separation algorithm to categorize voxels into three classes: background, pulmonary arteries, and pulmonary veins.

Firstly, the input CT images underwent preprocessing, including cropping, normalization, and resampling. Cropping was conducted based on the lung mask’s bounding box, yielding smaller images. These cropped images were then resampled to a target resolution of 0.726 mm, 0.726 mm, and 0.8 mm. The intensity values of the resampled images were clipped to 0.5%–99.5% of the foreground intensity value, followed by normalization using means ± Standard Deviations (SDs) of the foreground intensity value. Secondly, with a U-Net-based model, the preprocessed images underwent inference, and a sliding window algorithm was used to handle the varying volume sizes. The full volume was divided into blocks based on patch size and neural networks predicted outputs for each block, which were then scaled using a Gaussian function and summed up within overlapping regions. Finally, post-processing algorithms refined the results. Since the model inference used a cropped volume, a rollback geometry implementation was applied to resample resolution and reinsert the cropped volume into the original volume. This operation relied on the bounding box used for volume cropping during preprocessing. A post-processing algorithm, accessed *via* a C interface, performed outlier removal and adjusted the vessel branch for labeling the changes.

The algorithm automatically extracted IPVV from both lung and mediastinal windows. Two radiologists (with 4 and 15 years of experience in chest CT interpretation, respectively) visually evaluated all data. After their evaluation, any identified mislabeling of arteries and veins was manually corrected. Quantitative measurements of Total Intrapulmonary Vessel Volume (TIPVV), Intrapulmonary arterial Vessel Volume (IPVVa), Intrapulmonary venous Vessel Volume (IPVVv), and IPVV of small pulmonary vessels across various diameter categories (pulmonary vessels < 0.8 mm, 0.8 mm < pulmonary vessels < 1.6 mm, 1.6 mm < pulmonary vessels < 2.4 mm, 2.4 mm < pulmonary vessels < 3.2 mm, 3.2 mm < pulmonary vessels < 4.0 mm, pulmonary vessels > 4.0 mm) were obtained. Because pulmonary artery size is known to be associated with Body Surface Area (BSA) [23], each participant’s BSA was calculated to adjust the vascular parameters using a standard formula [24].

### 3.3 Statistical Analysis

All statistical analyses were performed using Statistical Package for the Social Sciences (SPSS) (version 26; IBM, New York, USA), and data analysis was performed using the GraphPad Prism software (version 9.5.1; GraphPad Prism software, San Diego, USA). Data have been presented as mean ± Standard Deviations (SDs) or as medians (interquartile range), while enumeration data have been expressed as percentage. Differences in these parameters were assessed using t-tests or U-tests, depending on the outcome of the normality test. A *p*-value of less than 0.05 (*p* < 0.05) was considered statistically significant.

## 4 RESULTS

### 4.1 Participant Characteristics

Based on the inclusion and exclusion criteria, 258 healthy participants were enrolled in this study. Their baseline characteristics are summarized in Table **1**. All participants underwent lung window and mediastinal window thin-slice (CT) scans. Among the participants, 116 were male (45%) and 142 were female (55%). The algorithm achieved an extraction success rate of approximately 100%.

### 4.2 Comparison of IPVV Extracted from Lung and Mediastinal Windows

**Fig. (4):**
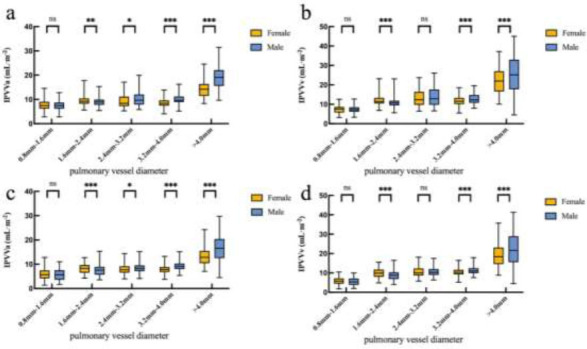
Intrapulmonary vessel volume adjusted for body surface area by diameter. (**a**, **b**) Lung window; (**c**, **d**) mediastinal window. IPVV, intrapulmonary vessel volume; ns, not significant; *, ** and ***, significant differences between males and females at *p* < 0.05, *p* <0.01, and *p* <0.001, respectively.

Fig. (**2**) illustrates the differences in IPVV extracted from the lung and mediastinal windows in axial, coronal, and three-dimensional (3D) images, underscoring the lung window’s advantage in displaying distal pulmonary small vessels. Table **2** shows that the TIPVV, IPVVa, IPVVv, and pulmonary small vessel volumes extracted from the lung window were significantly higher than those from the mediastinal window. Fig. (**3**) presents 3D reconstructions of IPVV in healthy individuals, with color-coded lung segments based on different diameters. No sex-based differences were found in pulmonary arteries and veins between 0.8 mm and 1.6 mm in either window. However, females exhibited significantly higher IPVV for pulmonary vessels between 1.6 mm and 2.4 mm. There were no statistically significant sex-based differences for pulmonary veins between 2.4 mm and 3.2 mm; however, males consistently showed higher values for all remaining IPVVs (Fig. **4**). Fig. (**5**) shows that IPVVs in both males and females decrease progressively with advancing age. Overall, our results have suggested TIPVV, IPVVa, and IPVVv to be generally greater in males than in females.

## 5 DISCUSSION

The distribution and diameter of small blood vessels in the lungs are indicative of pulmonary blood flow, which is essential for diagnosing intrapulmonary vascular disease. Over recent decades, CT technology has become invaluable for visualizing lung structures and quantifying pulmonary abnormalities. Modern CT scans, utilizing advanced imaging techniques, provide highly accurate representations of pulmonary anatomy, including the automated identification and extraction of pulmonary blood vessel measurements [7, 25]. CT scans also facilitate the examination of airway remodeling and changes in the pulmonary vasculature. In this study, IPVV was extracted and reconstructed from CT scans of healthy participants using both lung and mediastinal windows. The characteristics of pulmonary blood vessels with varying diameters were quantified to enhance an understanding of the morphological traits of the pulmonary vasculature. The objective of this study was to explore the potential of automated techniques in CT image analysis to evaluate IPVV and assess the impact of different window techniques on measurement outcomes. This study has provided normal value ranges for IPVV with different vascular diameters in a healthy population, aiming to identify appropriate measurement parameters. The findings have indicated that non-contrast CT scans could be utilized as a screening tool in the future, aiding in the early detection of small pulmonary vessel abnormalities in clinical diagnosis and treatment.

We have performed automatic extraction and reconstruction of all vascular structures across pulmonary tissue and quantified vessels in various lung regions. Generally, IPVV extracted from the lung window exceeded that from the mediastinal window. Together, arteries and veins accounted for approximately 50% of total pulmonary volume. No statistical differences were found between males and females in either IPVVa or IPVVv for vessels with diameters between 0.8–1.6 mm or for IPVVv for vessels with diameters of 2.4–3.2 mm. However, females showed higher average IPVVa and IPVVv than males for vessels with diameters between 1.6–2.4 mm. Conversely, the remaining IPVV was larger in males than in females, and this was consistent with prior research findings [1]. Specifically, Pienn *et al*. examined the number and volume of 2–10 mm pulmonary vessels during CT pulmonary angiography and demonstrated that males had more vascular segments than females across all vessel diameter ranges. This discrepancy may have likely risen from the fact that adult males generally have larger body sizes and higher cardiac output than adult females. Additionally, males possess larger pulmonary vascular trees to accommodate gas exchange, resulting in higher IPVV compared to females. Images tend to be smoother in mediastinal window reconstructions and sharper in bone-window reconstructions. Different window reconstructions facilitate varied disease diagnoses [26]. The lung window has been reported to demonstrate better specificity, precision, and sensitivity than the mediastinal window. Adjusting the window level and width has been found to improve differentiation between Ground-glass Opacities (GGOs) from bronchi and pulmonary blood vessels [27, 28]. Moreover, the lung window is more accurate than the mediastinal window for assessing tumor size and providing valuable information for predicting malignant tumors [29, 30]. This may be attributed to the default lung window settings, including a higher window width and lower window levels. The lung window enhances contrasts between pulmonary tissue and air, facilitating a clear visualization of delicate structures, like small pulmonary vessels. Conversely, the mediastinal window is better suited for observing various mediastinal structures.

Previous studies have demonstrated the percentage of small pulmonary vessels with a Cross-sectional Area (CSA) less than 5 mm^2^ as useful for assessing changes in pulmonary vasculature and determining the severity of Chronic Obstructive Pulmonary Disease (COPD) [31, 32]. Additionally, CSA measurements of small pulmonary vessels provide information on pulmonary hypertension [33], facilitating early disease detection and monitoring. Jacob *et al*. reported that measurements of pulmonary vessels and vessel-related structures using CT can enhance prognosis prediction in patients with Idiopathic Pulmonary Fibrosis (IPF) [34]. However, these assessments have been conducted using one-dimensional levels, introducing subjectivity and limiting comprehensive evaluations of intrapulmonary vasculature. In contrast, three-dimensional (3D) volumetry, employed in vascular systems and conditions, such as abdominal aortic aneurysms [35, 36], offers increased reliability by accurately reflecting morphological changes [37]. Furthermore, measurements of IPVV categorized by vessel size could provide critical insights into the progression of IPF. Previous studies have shown that the involvement of smaller vessels and changes in vessel morphology often serve as early indicators of disease progression in IPF, potentially reflecting early fibrotic changes. By quantifying IPVV and categorizing it by vessel size, clinicians may better stratify patients based on disease severity, monitor progression, and tailor management strategies accordingly. This study lays a foundational understanding of IPVV measurement in healthy individuals, paving the way for future research to validate these findings in patients with IPF and other interstitial lung diseases. This simple, replicable, and objective IPVV assessment method holds significant value.

Our algorithm automatically extracted IPVVs and differentiated between pulmonary arteries and veins. The results of the algorithm have been found to be objective and accurate. The segmentation and extraction methods we applied have been found to be efficient and rapid, suggesting that our algorithm could be applied and assessed in future large-sample studies. Additionally, Artificial Intelligence (AI) may provide further insights into medical imaging as it continues to evolve [38]. Despite our significant findings, this study has involved some limitations.

Firstly, as a single-center, retrospective study with a small sample size, our healthy participants may not represent the broader Chinese population. Secondly, selection bias was present and, thirdly, the size estimations for pulmonary blood vessels were limited by CT pixel size (~0.8 mm), which made it impossible to capture the pulmonary microvasculature (estimated capillary blood volume was 140 mL) [2]. However, a previous study has reported that vessels with a diameter less than 1.2 mm have a stronger correlation with survival prediction than those with a diameter less than 0.8 mm [16]. This may be because although small vessels indicate microvascular damage, they contribute little to the total vascular volume. In contrast, larger vessels tend to be more clinically relevant to functional outcomes and are more reflective of overall health. Currently, most conventional CT scanners operate with a 512 matrix, and the FOV for chest scans is typically between 350-400 mm. The algorithm used in this study can be applied to most CT scanners. In the future, with the development and widespread acceptance of 1024 matrix machines, pixel values and thresholds will be significantly reduced. This study has thus established a foundation and offered guidance for the future use of non-contrast CT scans as a potential screening tool for pulmonary vascular diseases, aiming to facilitate the early detection of pulmonary vascular abnormalities in clinical practice.

## CONCLUSION

Herein, a quantitative analysis algorithm has been developed to enable the automatic extraction of IPVV from plain chest CT scans. Our findings have indicated pulmonary small vessels to be more visible in the lung window, compared to the mediastinal window, providing higher accuracy in quantifying these structures.

## Figures and Tables

**Fig. (1):**
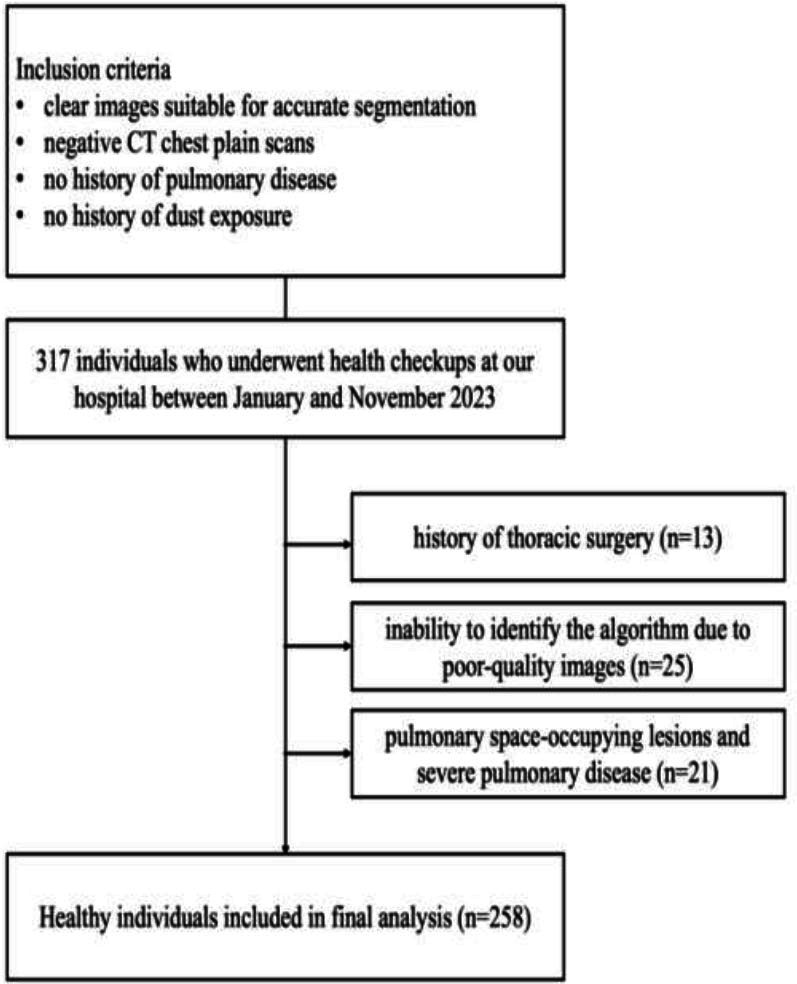
Flow diagram of the included individuals.

**Fig. (2):**
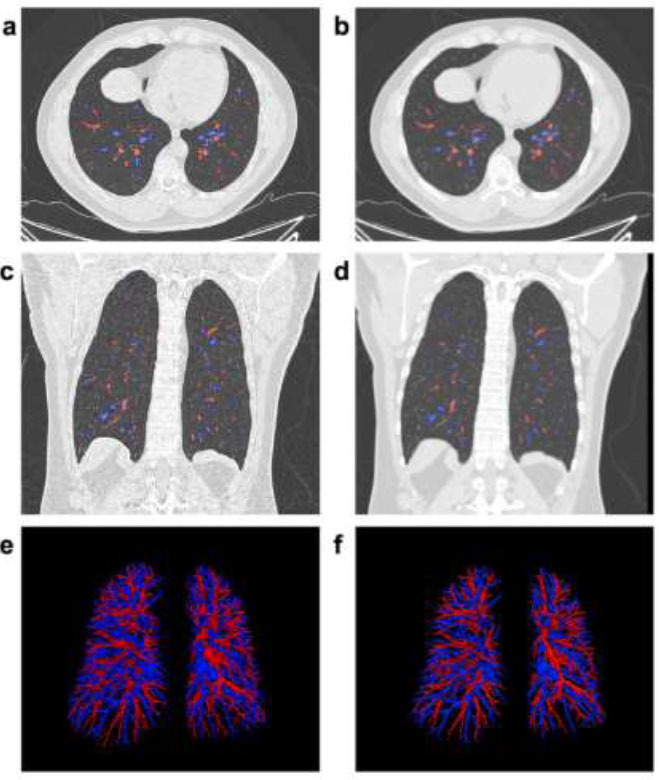
Differences between the lung window and the mediastinal window in axial, coronal, and 3D images. (**a**) Automatic annotation of pulmonary arteries and veins in the transverse plane of a male participant based on lung window images. (**b**) Mediastinal window images adjusted to match the lung’s window width and level, highlighting the differences between the two windows. (**c**, **d**) Images in the coronal plane. (**e**, **f**) Intrapulmonary vessel volume extracted using both lung and mediastinal windows. Arteries are marked in red and veins are marked in blue.

**Fig. (3):**
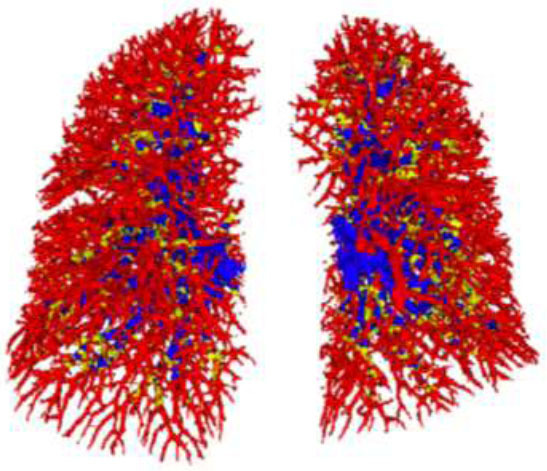
Blood vessel sizes indicated by different colors; 0.8 mm< pulmonary vessels <3.2 mm are marked in red, 3.2 mm< pulmonary vessels <4.0 mm are indicated in yellow, and pulmonary vessels >4.0 mm are indicated in blue.

**Fig. (5):**
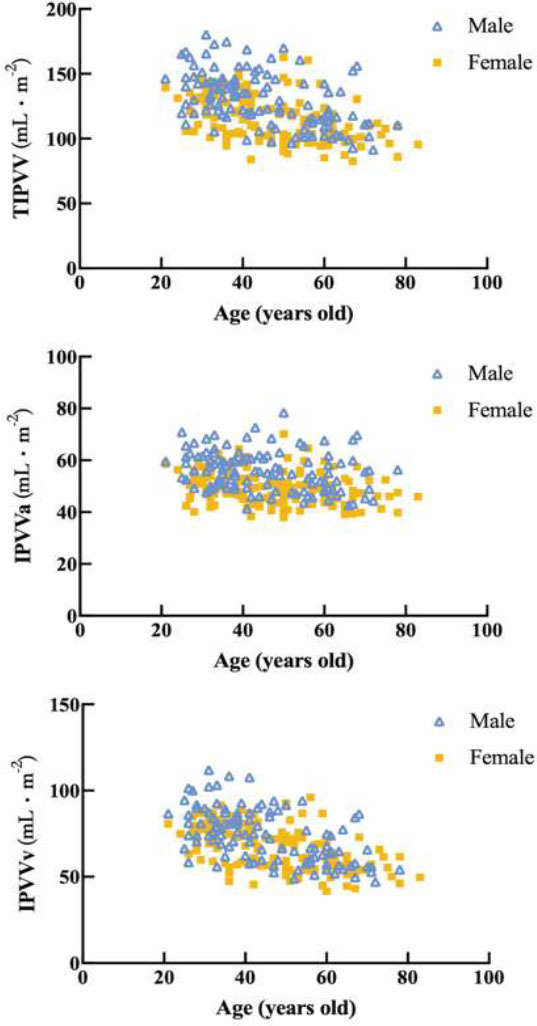
Relationship between age and TIPVV, IPVVa, and IPVVv in the lung window. IPVV, intrapulmonary vessel volume; TIPVV, total intrapulmonary vessel volume; IPVVa, intrapulmonary arterial vessel volume; IPVVv, intrapulmonary venous vessel volume.

**Table 1:** Baseline characteristics of the study sample.

Healthy individuals (n=258)	
Age, years (median, IQR)	44 (35, 57)
Sex	-
Female	142 (55)
Male	116 (45)
Weight, kg (median, IQR)	65.75 (58, 74)
Height, cm (median, IQR)	168 (161,173)

**Note: **Categorical data have been presented as numbers with percentages in parentheses. IQR, interquartile.

**Table 2:** Comparison of IPVV between the lung and mediastinal windows.

IPVV	Lung Window	Mediastinal Window	t/Z
TIPVV, mL·m^-2^	120.42 (105.19, 138.08)	102.33 (92.82, 112.23)	-10.831***
IPVVa, mL·m^-2^	51.28 (46.48, 57.54)	44.67 (40.83, 49.46)	-9.884***
IPVVv, mL·m^-2^	69.08 (57.74, 81.12)	57.10 (51.25, 64.28)	-9.919***
IPVVa, mL·m^-2^	-	-	-
pulmonary vessels <0.8 mm	-	-	-
0.8 mm< pulmonary vessels <1.6 mm	7.44 (6.17, 8.92)	5.61 (3.84, 7.36)	-8.709***
1.6 mm< pulmonary vessels <2.4 mm	9.02 (8.01, 10.09)	7.69 (5.85, 8.98)	-7.991***
2.4 mm< pulmonary vessels <3.2 mm	9.08 (7.49, 11.57)	7.83 (6.71, 9.22)	-6.589***
3.2 mm< pulmonary vessels <4.0 mm	9.05 (7.88, 10.14)	8.29 (7.13, 9.48)	-4.324***
pulmonary vessels >4.0 mm	16.09 (13.19, 19.78)	14.86 (11.86, 18.55)	-3.069**
IPVVv, mL·m^-2^	-	-	-
pulmonary vessels <0.8 mm	-	-	-
0.8 mm< pulmonary vessels <1.6 mm	7.29±1.91	5.55±1.94	10.289***
1.6 mm< pulmonary vessels <2.4 mm	11.05 (9.94, 12.65)	9.20 (7.24, 10.82)	-9.338***
2.4 mm< pulmonary vessels <3.2 mm	13.01 (9.99, 17.51)	10.23 (8.84, 12.30)	-7.630***
3.2 mm< pulmonary vessels <4.0 mm	12.04 (10.69, 14.16)	10.68 (9.51, 12.05)	-7.029***
pulmonary vessels >4.0 mm	24.00 (18.41, 30.82)	20.57 (16.03, 25.95)	-4.934***

**Note:** Normally distributed data are presented as means ± standard deviations, while data not conforming to normal distributions are expressed as median (lower quartile, upper quartile). IPVV, intrapulmonary vessel volume; TIPVV, total intrapulmonary vessel volume; IPVVa, intrapulmonary arterial vessel volume; IPVVv, intrapulmonary venous vessel volume. ** and *** indicate significant differences between the lung window and mediastinal window at *p* <0.01 and *p* <0.001.

## Data Availability

The data of current study are available from author, [X.S.] and [W.S.], on a reasonable request.

## LIST OF ABBREVIATIONS

BSA: Body surface area
COPD: Chronic obstructive pulmonary disease
COVID-19: Coronavirus disease 2019
CSA: Cross-sectional area
CT: Computed tomography
DICOM: Digital Imaging and Communications in Medicine
GGOs: Ground-glass opacities
IPVV: Intrapulmonary vessel volume
IPPVa: Intrapulmonary arterial vessel volume
IPPVv: Intrapulmonary venous vessel volume
TIPVV: Total intrapulmonary vessel volume

## STANDARDS OF REPORTING

STROBE guidelines were followed.

## References

[1] Pienn M., Burgard C., Payer C., Avian A., Urschler M., Stollberger R., Olschewski A., Olschewski H., Johnson T., Meinel F.G., Bálint Z. (2018). Healthy lung vessel morphology derived from thoracic computed tomography.. Front. Physiol..

[2] Aaron C.P., Hoffman E.A., Lima J.A.C., Kawut S.M., Bertoni A.G., Vogel-Claussen J., Habibi M., Hueper K., Jacobs D.R., Kalhan R., Michos E.D., Post W.S., Prince M.R., Smith B.M., Ambale-Venkatesh B., Liu C.Y., Zemrak F., Watson K.E., Budoff M., Bluemke D.A., Barr R.G. (2017). Pulmonary vascular volume, impaired left ventricular filling and dyspnea: The MESA Lung Study.. PLoS One.

[3] Huang W, Yen RT, McLaurine M, Bledsoe G (1996). Morphometry of the human pulmonary vasculature.. J Appl Physiol.

[4] Horsfield K., Gordon W.I. (1981). Morphometry of pulmonary veins in man.. Lung.

[5] Singhal S., Henderson R., Horsfield K., Harding K., Cumming G. (1973). Morphometry of the human pulmonary arterial tree.. Circ. Res..

[6] Sluimer I., Schilham A., Prokop M., van Ginneken B. (2006). Computer analysis of computed tomography scans of the lung: A survey.. IEEE Trans. Med. Imaging.

[7] Nardelli P., Jimenez-Carretero D., Bermejo-Pelaez D., Washko G.R., Rahaghi F.N., Ledesma-Carbayo M.J., San Jose Estepar R. (2018). Pulmonary artery-vein classification in CT images using deep learning.. IEEE Trans. Med. Imaging.

[8] Resten A., Maitre S., Musset D. (2005). CT imaging of peripheral pulmonary vessel disease.. Eur. Radiol..

[9] Marano R., Pirro F., Silvestri V., Merlino B., Savino G., Rutigliano C., Meduri A., Natale L., Bonomo L. (2015). Comprehensive CT cardiothoracic imaging: A new challenge for chest imaging.. Chest.

[10] Sato Y., Nakajima S., Shiraga N., Atsumi H., Yoshida S., Koller T., Gerig G., Kikinis R. (1998). Three-dimensional multi-scale line filter for segmentation and visualization of curvilinear structures in medical images.. Med. Image Anal..

[11] Frangi A.F., Niessen W.J., Hoogeveen R.M., van Walsum T., Viergever M.A. (1999). Model-based quantitation of 3-D magnetic resonance angiographic images.. IEEE Trans. Med. Imaging.

[12] Lesage D., Angelini E.D., Bloch I., Funka-Lea G. (2009). A review of 3D vessel lumen segmentation techniques: Models, features and extraction schemes.. Med. Image Anal..

[13] Shahin Y., Alabed S., Alkhanfar D., Tschirren J., Rothman A.M.K., Condliffe R., Wild J.M., Kiely D.G., Swift A.J. (2022). Quantitative CT evaluation of small pulmonary vessels has functional and prognostic value in pulmonary hypertension.. Radiology.

[14] van Rikxoort E.M., van Ginneken B. (2013). Automated segmentation of pulmonary structures in thoracic computed tomography scans: A review.. Phys. Med. Biol..

[15] Sun X., Meng X., Zhang P., Wang L., Ren Y., Xu G., Yang T., Liu M. (2022). Quantification of pulmonary vessel volumes on low-dose computed tomography in a healthy male Chinese population: The effects of aging and smoking.. Quant. Imaging Med. Surg..

[16] Alkhanfar D., Shahin Y., Alandejani F., Dwivedi K., Alabed S., Johns C., Lawrie A., Thompson A.A.R., Rothman A.M.K., Tschirren J., Uthoff J.M., Hoffman E., Condliffe R., Wild J.M., Kiely D.G., Swift A.J. (2022). Severe pulmonary hypertension associated with lung disease is characterised by a loss of small pulmonary vessels on quantitative computed tomography.. ERJ Open Res..

[17] Melzig C., Wörz S., Egenlauf B., Partovi S., Rohr K., Grünig E., Kauczor H.U., Heussel C.P., Rengier F. (2019). Combined automated 3D volumetry by pulmonary CT angiography and echocardiography for detection of pulmonary hypertension.. Eur. Radiol..

[18] Huang X., Yin W., Shen M., Wang X., Ren T., Wang L., Liu M., Guo Y. (2022). Contributions of emphysema and functional small airway disease on intrapulmonary vascular volume in COPD.. Int. J. Chron. Obstruct. Pulmon. Dis..

[19] Cho Y.H., Lee S.M., Seo J.B., Kim N., Bae J.P., Lee J.S., Oh Y.M., Do-Lee S. (2018). Quantitative assessment of pulmonary vascular alterations in chronic obstructive lung disease: Associations with pulmonary function test and survival in the KOLD cohort.. Eur. J. Radiol..

[20] Lins M., Vandevenne J., Thillai M., Lavon B.R., Lanclus M., Bonte S., Godon R., Kendall I., De Backer J., De Backer W. (2020). Assessment of small pulmonary blood vessels in COVID-19 patients using HRCT.. Acad. Radiol..

[21] Dierckx W, De Backer W, Lins M (2022). CT-derived measurements of pulmonary blood volume in small vessels and the need for supplemental oxygen in COVID-19 patients.. J Appl Physiol.

[22] Ratanawatkul P., Oh A., Richards J.C., Swigris J.J. (2020). Performance of pulmonary artery dimensions measured on high-resolution computed tomography scan for identifying pulmonary hypertension.. ERJ Open Res..

[23] Karazincir S., Balci A., Seyfeli E., Akoğlu S., Babayiğit C., Akgül F., Yalçin F., Eğilmez E. (2008). CT assessment of main pulmonary artery diameter.. Diagn. Interv. Radiol..

[24] Du Bois D., Du Bois E.F. (1989). A formula to estimate the approximate surface area if height and weight be known. 1916.. Nutrition.

[25] Payer C., Pienn M., Bálint Z., Shekhovtsov A., Talakic E., Nagy E., Olschewski A., Olschewski H., Urschler M. (2016). Automated integer programming based separation of arteries and veins from thoracic CT images.. Med. Image Anal..

[26] Lu Z., Long F., He X. (2022). Classification and segmentation algorithm in benign and malignant pulmonary nodules under different CT reconstruction.. Comput. Math. Methods Med..

[27] Bak S.H., Lee H.Y., Kim J.H., Um S.W., Kwon O.J., Han J., Kim H.K., Kim J., Lee K.S. (2016). Quantitative CT scanning analysis of pure ground-glass opacity nodules predicts further CT scanning change.. Chest.

[28] Yao G. (2016). Value of window technique in diagnosis of the ground glass opacities in patients with non-small cell pulmonary cancer.. Oncol. Lett..

[29] Grills I.S., Fitch D.L., Goldstein N.S., Yan D., Chmielewski G.W., Welsh R.J., Kestin L.L. (2007). Clinicopathologic analysis of microscopic extension in lung adenocarcinoma: Defining clinical target volume for radiotherapy.. Int. J. Radiat. Oncol. Biol. Phys..

[30] Lu H., Kim J., Qi J., Li Q., Liu Y., Schabath M.B., Ye Z., Gillies R.J., Balagurunathan Y. (2020). Multi-window CT based radiological traits for improving early detection in lung cancer screening.. Cancer Manag. Res..

[31] Matsuoka S., Washko G.R., Dransfield M.T., Yamashiro T., San Jose Estepar R., Diaz A., Silverman E.K., Patz S., Hatabu H. (2010). Quantitative CT measurement of cross-sectional area of small pulmonary vessel in COPD: correlations with emphysema and airflow limitation.. Acad. Radiol..

[32] Takayanagi S., Kawata N., Tada Y., Ikari J., Matsuura Y., Matsuoka S., Matsushita S., Yanagawa N., Kasahara Y., Tatsumi K. (2017). Longitudinal changes in structural abnormalities using MDCT in COPD: Do the CT measurements of airway wall thickness and small pulmonary vessels change in parallel with emphysematous progression?. Int. J. Chron. Obstruct. Pulmon. Dis..

[33] Matsuoka S., Washko G.R., Yamashiro T., Estepar R.S.J., Diaz A., Silverman E.K., Hoffman E., Fessler H.E., Criner G.J., Marchetti N., Scharf S.M., Martinez F.J., Reilly J.J., Hatabu H. (2010). Pulmonary hypertension and computed tomography measurement of small pulmonary vessels in severe emphysema.. Am. J. Respir. Crit. Care Med..

[34] Jacob J., Bartholmai B.J., Rajagopalan S., van Moorsel C.H.M., van Es H.W., van Beek F.T., Struik M.H.L., Kokosi M., Egashira R., Brun A.L., Nair A., Walsh S.L.F., Cross G., Barnett J., de Lauretis A., Judge E.P., Desai S., Karwoski R., Ourselin S., Renzoni E., Maher T.M., Altmann A., Wells A.U. (2018). Predicting outcomes in idiopathic pulmonary fibrosis using automated computed tomographic analysis.. Am. J. Respir. Crit. Care Med..

[35] Kauffmann C., Tang A., Therasse É., Giroux M.F., Elkouri S., Melanson P., Melanson B., Oliva V.L., Soulez G. (2012). Measurements and detection of abdominal aortic aneurysm growth: Accuracy and reproducibility of a segmentation software.. Eur. J. Radiol..

[36] Kontopodis N., Metaxa E., Papaharilaou Y., Georgakarakos E., Tsetis D., Ioannou C.V. (2014). Value of volume measurements in evaluating abdominal aortic aneurysms growth rate and need for surgical treatment.. Eur. J. Radiol..

[37] Rengier F., Wörz S., Melzig C., Ley S., Fink C., Benjamin N., Partovi S., von Tengg-Kobligk H., Rohr K., Kauczor H.U., Grünig E. (2016). Automated 3D volumetry of the pulmonary arteries based on magnetic resonance angiography has potential for predicting pulmonary hypertension.. PLoS One.

[38] Dwivedi K., Sharkey M., Condliffe R., Uthoff J.M., Alabed S., Metherall P., Lu H., Wild J.M., Hoffman E.A., Swift A.J., Kiely D.G. (2021). Pulmonary hypertension in association with lung disease: Quantitative CT and artificial intelligence to the rescue? State-of-the-Art rev.. Diagnostics.

