# Supplementary material for: Automated 3D Quantitative Analysis of Intrapulmonary Vessel Volume on Non-contrast CT in Healthy Individuals
Source: Curr Med Imaging. 2025 Jan 24;21:e15734056354924. doi: 10.2174/0115734056354924241115102310 (PMC12817174; doi:10.2174/0115734056354924241115102310)
Supplement: Supplementary file 1 — Supplementary material is available on the Publisher’s website. [file CMIM-21-E15734056354924_SD1.pdf]

1573-4056/25

Send Orders for Reprints to reprints@benthamscience.net

BENTHAM SCIENCE

CrossMark

Current Medical Imaging

Content list available at: https://benthamscience.com/journals/cmir

Supplementary Material

Automated 3D Quantitative Analysis of Intrapulmonary Vessel Volume on Non-contrast CT in Healthy Individuals

Ying Ming[1], Yu Zhang[2], Ran Xiao[1], Ruijie Zhao[1], Jiaru Wang[1], Sirong Piao[1], Lan Song[1], Yinghao Xu[3], Xin Sui[1,*] and Wei Song[1,*]

[1]*Department of Radiology, Peking Union Medical College Hospital, Chinese Academy of Medical Sciences and Peking Union Medical College, Beijing 100730, China*
[2]*Research and Development Center, Canon Medical Systems (China), No.3, Xinyuan South Road, Chaoyang District, Beijing 100027, China*
[3]*CT Business Unit, Canon Medical Systems (China), No.3, Xinyuan South Road, Chaoyang District, Beijing 100027, China*

Design and Evaluation for Pulmonary Vessel Segmentation and Separation
Oct. 2024
CANON
(CMC / RDC)

1. ALGORITHM DESIGN FOR PULMONARY VESSEL SEGMENTATION

1.1. Overall Pipeline

The pulmonary artery/vein separation algorithm is implemented to classify voxels into three classes: background, pulmonary arteries and pulmonary veins. A total of 440 datasets are annotated to train a U-Net based vessel segmentation model. The input CT images are firstly pre-processed through cropping, normalization, and resampling. Then the pre-processed images are inferred by the model and the results are refined by a post-processing algorithm.

1.2. Data Sets

There are 440 datasets used to develop the model (training and test: 370/70) including 219 non-contrast cases.

- The datasets are from different vendors: Canon/GE/Siemens/Philips
- Including both private datasets (collected from different countries) and public datasets (LIDC-IDRI, https://www.cancerimagingarchive.net/collection/lidc-idri/)
- The dataset covers normal population, lung nodule patients, and other patients without significant damage to the pulmonary vascular structure

1.3. Annotation Procedure

The annotation doctors have more than 5 years of work experience in thorax CT.

The annotation process includes four steps.

a) Pre-segmentations are generated using a coarse-stage pulmonary vessel segmentation model.

b) Doctors refine the vessel mask of the pre-segmentations in step and append missing small branches. The target small branches are those with a diameter larger than 1.5mm.

c) The vessel is automatically extended to the very small branches based on refined results in step b) by region growing. The target for region growing is to extend vessels with a diameter of 1mm.

d) Doctors check and correct errors in the above extended results.

DOI: 10.2174/0115734056354924241115102310, 2025, *21*, e15734056354924

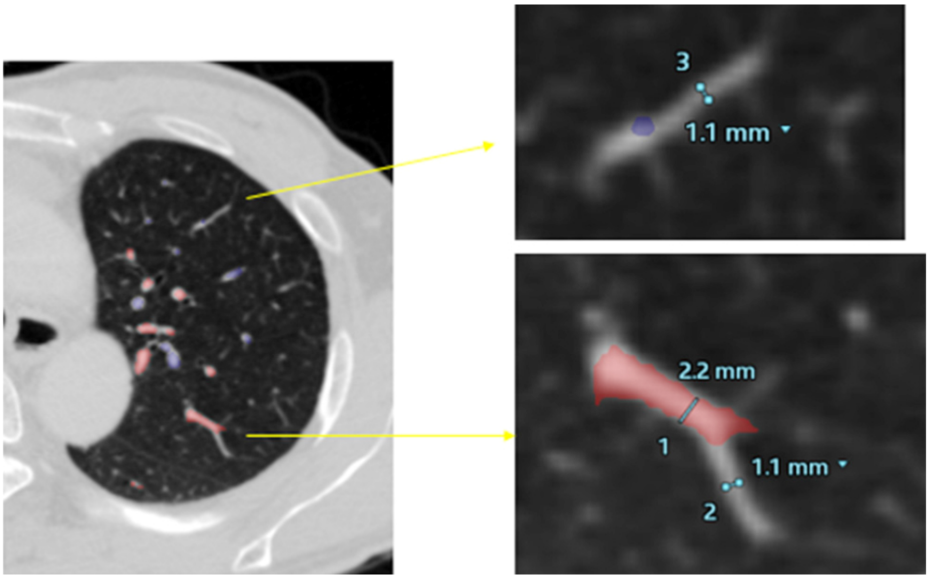

The image shows the vessels of different diameters.

1.4. Pre-processing Algorithm Design

The pre-processing includes the following steps:

a. The CT images are cropped into smaller images according to the bounding box of their lung masks.

b. The cropped images are resampled into the target resolution (0.726mm,0.726mm, 0.8mm) in patient X, Y, and Z dimensions.

c. The intensity values of the resampled images are clipped into the range of 0.5% to 99.5% of the image foreground intensity value, and then normalized according to the mean and the standard deviation of the image foreground intensity values.

1.5. Model Design

The network used in this algorithm is based on U-Net. The network architecture is as following:

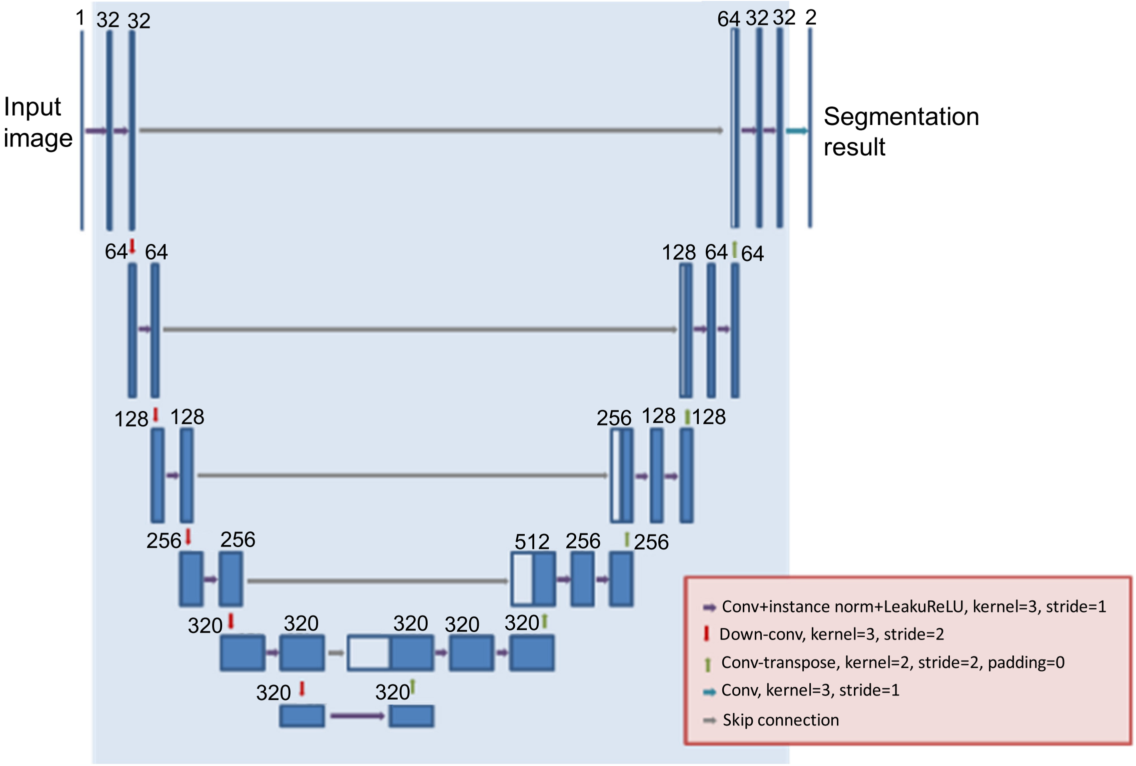

√ *Network structure*

• *U-Net*

• *3D*

• *17 convolutional layers*

• *Base channel 32*

• *30.8 M parameters*

√ *Network pruning: cut least 50% of channels for every convolutional layer*

√ *Framework: PyTorch*

√ *Loss function:*

• *Loss=CE loss+ Dice loss+0.5clDice loss*

• *CrossEntropyloss* $(y,\hat{y}) = -\sum_x (p(x)\log q(x))$

• *DiceLoss*$(y, \hat{y}) = 1-\frac{(2y\hat{y}+1)}{(y+\hat{y}+1)}$

• *Cldice loss introduced in [1]*

√ *Optimizer: adam*

√ *Learning rate and decay: If the training loss is not reduced over 30 epochs, the learning rate will decay to 0.2lr*

√ *Patch size: [192,256,96]*

√ *Batch: 1 (inference)*

To handle varying volume sizes, a sliding window algorithm is used. The full volume is split into blocks based on the patch size, and the neural network predicts on each block. The network outputs are scaled according to a Gaussian function and summed in the overlapping regions, meaning that voxels near the centre of a patch contribute more to the eventual classification than those near the edge of a patch.

Once the network outputs have been summed, the probability of each class for each voxel is calculated and then probability map is output.

1.6. Post-processing Algorithm Design

Due to the cropped volume is used for model inference, a rollback geometry implementation is necessary to resample the resolution and fill the cropped volume back to original volume. This operation relies on the bounding box during cropping in the pre-processing step. The highest likelihood class for each voxel is assigned as the predicted class for the voxel.

2. EVALUATION OF THE PERFORMANCE

2.1. Metrics

**Performance Metrics**	**Evaluation Results**
Criterion1:<br>Vessel diameter	The branches whose diameter ≥1mm can be segmented. It is confirmed by visual checking.
Criterion2:<br>Success rate (per case)	The match ratio of detected vessel path is 93%.
Criterion3:<br>Dice, centerline dice	• On 34 non-contrast test data<br>o Average artery domain dice with GT: 0.90<br>o Average vein domain dice with GT: 0.89<br>o Average artery centerline dice with GT: 0.94<br>o Average vein centerline dice with GT: 0.92<br>• On 36 contrast test data<br>o Average artery domain dice with GT: 0.87<br>o Average vein domain dice with GT: 0.85<br>o Average artery centerline dice with GT: 0.88<br>o Average vein centerline dice with GT: 0.90

* Centerline dice: the dice value is calculated based on centerline, not based on whole vessel mask [1].

2.2. Diagram and Visualization

• There are 34 non-contrast test cases. Artery dice, vein dice, artery centerline dice, vein centerline dice are shown in the figures.

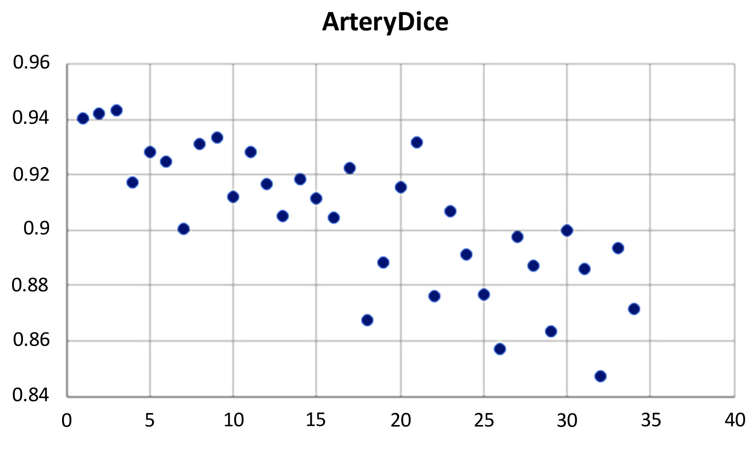

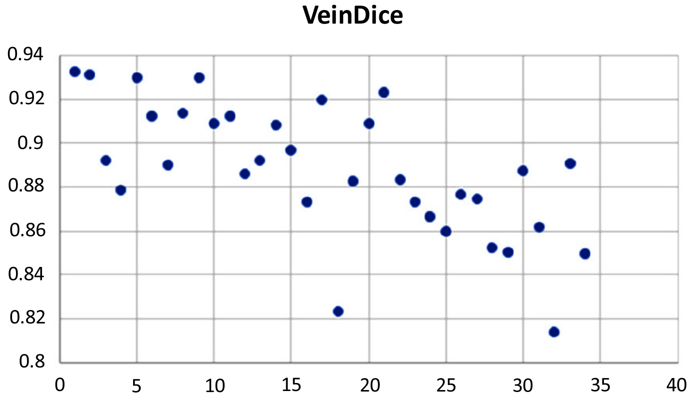

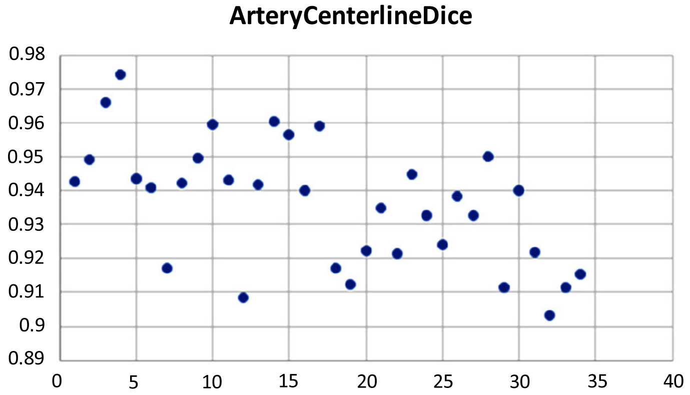

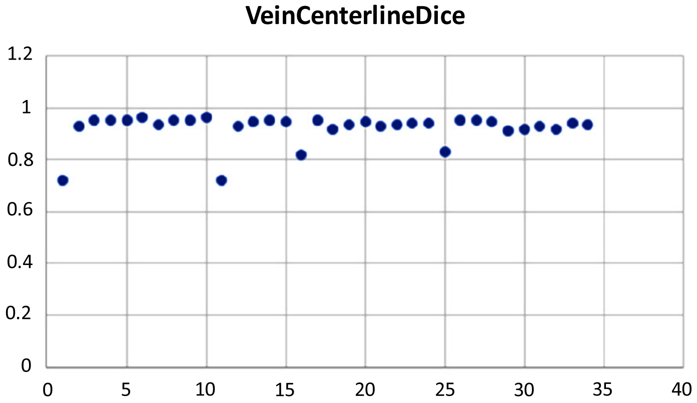

• Visualization

Blue is vein GT, green is vein prediction; Red is artery GT, purple is artery prediction

In the following captured images, the 1st line is the GT in upper of two domains, the 2nd line is the prediction in upper of two domains.

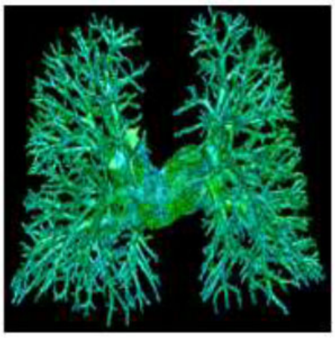

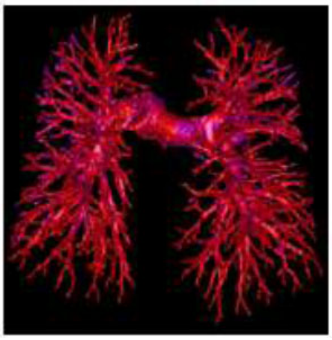

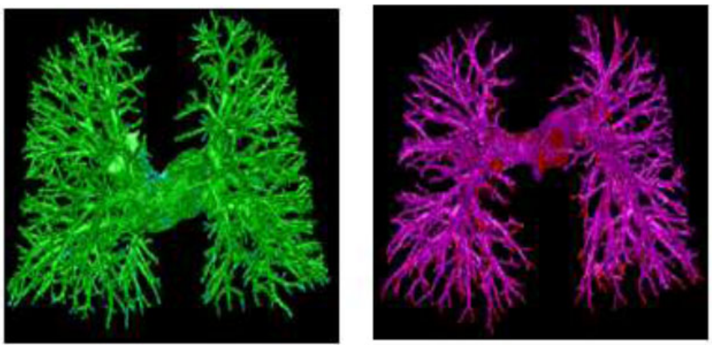

2.3. Limitations

The suggested slice thickness is ≤1.25mm, if slice thickness is larger than this, it may not achieve the expected accuracy.

Pulmonary embolism will not be ruled out and will be included in the vascular results.

This algorithm is not recommended for situations where large areas of consolidation or atelectasis exist in the lungs.

REFERENCES

[1] Shit S, Paetzold JC, Sekuboyina A, *et al.* clDice-a novel topology-preserving loss function for tubular structure segmentation[C]//Proceedings of the IEEE/CVF Conference on Computer Vision and Pattern Recognition. 2021; 16560-9.

© 2025 The Author(s). Published by Bentham Science Publisher.

This is an open access article distributed under the terms of the Creative Commons Attribution 4.0 International Public License (CC-BY 4.0), a copy of which is available at: https://creativecommons.org/licenses/by/4.0/legalcode. This license permits unrestricted use, distribution, and reproduction in any medium, provided the original author and source are credited.
